# NF-κB targeting by way of IKK inhibition sensitizes lung cancer cells to adenovirus delivery of TRAIL

**DOI:** 10.1186/1471-2407-10-584

**Published:** 2010-10-27

**Authors:** Cigdem Aydin, Ahter D Sanlioglu, Atil Bisgin, Burcak Yoldas, Levent Dertsiz, Bahri Karacay, Thomas S Griffith, Salih Sanlioglu

**Affiliations:** 1 Department of Medical Biology and Genetics, Human Gene and Cell Therapy Center of Akdeniz University Hospitals and Clinics, Antalya, 07058, Turkiye; 2 Department of Medical Genetics, Human Gene and Cell Therapy Center of Akdeniz University Hospitals and Clinics, Antalya, 07058, Turkiye; 3 Human Gene and Cell Therapy Center of Akdeniz University Hospitals and Clinics, Antalya, 07058, Turkiye; 4 Department of Thorasic Surgery, Akdeniz University Faculty of Medicine, Antalya, 07058, Turkiye; 5 Department of Pediatrics and Gene Therapy Center, University of Iowa, Iowa City, IA, 52242, USA; 6 Department of Urology and Gene Therapy Center, University of Iowa, Iowa City, IA, 52242, USA

## Abstract

**Background:**

Lung cancer causes the highest rate of cancer-related deaths both in men and women. As many current treatment modalities are inadequate in increasing patient survival, new therapeutic strategies are required. TNF-related apoptosis-inducing ligand (TRAIL) selectively induces apoptosis in tumor cells but not in normal cells, prompting its current evaluation in a number of clinical trials. The successful therapeutic employment of TRAIL is restricted by the fact that many tumor cells are resistant to TRAIL. The goal of the present study was to test a novel combinatorial gene therapy modality involving adenoviral delivery of TRAIL (Ad5hTRAIL) and IKK inhibition (AdIKKβKA) to overcome TRAIL resistance in lung cancer cells.

**Methods:**

Fluorescent microscopy and flow cytometry were used to detect optimum doses of adenovirus vectors to transduce lung cancer cells. Cell viability was assessed via a live/dead cell viability assay. Luciferase assays were employed to monitor cellular NF-κB activity. Apoptosis was confirmed using Annexin V binding.

**Results:**

Neither Ad5hTRAIL nor AdIKKβKA infection alone induced apoptosis in A549 lung cancer cells, but the combined use of Ad5hTRAIL and AdIKKβKA significantly increased the amount of A549 apoptosis. Luciferase assays demonstrated that both endogenous and TRAIL-induced NF-κB activity was down-regulated by AdIKKβKA expression.

**Conclusions:**

Combination treatment with Ad5hTRAIL and AdIKKβKA induced significant apoptosis of TRAIL-resistant A549 cells, suggesting that dual gene therapy strategy involving exogenous TRAIL gene expression with concurrent IKK inhibition may be a promising novel gene therapy modality to treat lung cancer.

## Backround

Lung cancer is the leading cause of cancer mortality in the world (31% for men and 26% for women of all cancer deaths) [1]. Despite the use of conventional multimodal treatment methods (chemotherapy, radiation, and surgery), the overall survival rate from lung cancer has improved little, with < 15% of patients surviving > 5 years [2]. Consequently, new therapeutic strategies, such as gene therapy, are being tested in preclinical and clinical settings. Knowing that apoptosis is a key mechanism in the regulation of tissue homeostasis, several members of the tumor necrosis factor (TNF) superfamily have been implicated in the process. TNF-related apoptosis-inducing ligand (TRAIL), also known as Apo2L, was originally identified through its homology to TNF, FasL, and other members of the TNF superfamily [3,4]. Like most other members of the TNF superfamily of ligands, TRAIL is primarily expressed as a type II membrane protein of 33-35 kD [5]. To date, four human membrane-bound receptors for TRAIL have been identified: DR4/TRAIL-R1, DR5/TRAIL-R2/KILLER, TRID/DcR1/TRAIL-R3, and DcR2/TRAIL-R4. Two of the membrane receptors, DR4 and DR5, contain the essential cytoplasmic death domain through which TRAIL can transmit an apoptotic signal. DcR1 and DcR2 can also bind TRAIL, but they appear to act as antagonistic receptors because they lack a functional death domain [6-9].

There are several reasons why TRAIL is of interest for people working on cancer gene therapy. TRAIL is unique in that it selectively induces apoptosis in tumor and transformed cells, but does not harm normal cells [10,11]. In addition, apoptosis induction in response to most DNA-damaging drugs usually requires functional tumor supressor p53 gene [12]. Because of the inactivation of p53 in more than 50% of human cancers during tumorigenesis, the tumors eventually display resistance to both radiotherapy and chemotherapy. TRAIL, however, can induce p53-independent apoptosis of cancer cells [13]. Despite this fact, a significant proportion of tumor cells display TRAIL resistance by a mechanism that is not yet fully understood [14,15]. Resistance to TRAIL-induced apoptosis, both in normal and cancer cells, was initially considered to be due to DcR1 and/or DcR2 expression, which compete with DR4 and DR5 for binding to TRAIL [6,16]. Apart from TRAIL receptor composition, [17,18] there are a number of other possible reasons why some cancer cells exhibit TRAIL resistance. For example, the presence of intracellular apoptosis inhibitory proteins (Bcl-xL, c-FLIP, cIAP etc.) or the loss of Bax and Bak function may lead to a TRAIL-resistant phenotype [14,19]. Interestingly, the engagement of DR4, DR5, and DcR2 can activate the NF-κB pathway [20,21], and high levels of endogenous NF-κB activity interfere with TRAIL-induced apoptosis. Thus, targeting the NF-κB signaling pathway may help sensitize cancer cells to TRAIL. In this study, a complementary gene therapy modality using adenovirus-mediated delivery of an IKKβΚA mutant (AdIKKβKA) was deployed to test the extent to which NF-κB inhibition sensitized lung cancer cells to TRAIL (Ad5hTRAIL).

## Methods

### Adenovirus Preparation

Recombinant adenoviral vectors, AdEGFP [22], Ad5hTRAIL [23], AdIKKβKA [24], AdNFκBLuc [25], and AdCMVLacZ [26,27], were amplified in 293 cells and purified by cesium chloride gradient. After vector purification, adenoviral vectors were kept at -80°C in 10 m*M *Tris containing 20% glycerol. The titers of purified adenoviral stocks were measured to be 10^13 ^DNA particles/ml. AdIKKβKA encodes the dominant negative mutant form (K44A) of IKKβ and forms inactive IKK complex so that IKKβ does not phosphorylate IkB. IkBαSR produces dominant negative mutant form (S32A/S36A) of IkBα. Thus, the IKK complex cannot phosphorylate mutant IkBα from S32 and S36 residues. By doing so NF-κB is always sequestered in cytoplasm. Both mutant proteins interfere with NF-κB signaling at different levels of the signaling cascade.

### Cell Culture

The human non-small cell lung carcinoma cell line A549 was obtained from American Type Culture Collection. Cells were cultured in RPMI 1640 medium supplemented with 10% FBS, 2.2 g/l sodium bicarbonate, 1 mM L-glutamine, and 1% penicillin-streptomycin-amphoterisine mixture (PSA) using Thermo SteriCult incubators. The study was carried out in accordance with Declaration of Helsinki and approved by the Akdeniz University Committee on Ethics.

### Adenoviral Infection of Lung Cancer Cells

Cells were cultured and permitted to adhere for at least 24 hr before adding adenovirus vectors. Before the infection, lung cancer cells were washed with PBS, and then infected with vectors at increasing multiplicity of infection (MOI). Cells were first kept at 37°C in RPMI 1640 medium without FBS for 2 h. An equal volume of RPMI 1640 supplemented with 20% FBS was then added to cells. To measure transduction efficiency, the percentage of EGFP^+ ^cells was determined by using fluorescent microscopy and flow cytometry 48 h after infection. The cell viability was assessed using Propidium iodide exclusion technique.

### Cell Viability Assay

Live/Dead Cellular Viability/Cytotoxicity Kit (Molecular Probes; Eugene, OR) was used to discriminate live cells from dead cells. This assay is based on the use of Calsein AM and Ethidium homodimer-1 (EthD-1). Calsein AM is a fluorogenic substrate for intracellular calsein esterase. It is modified to a green fluorescent compound (calsein) by active esterase in live cells with intact membranes. In addition, live cells do not allow EthD-1, a red fluorescent nucleic acid stain, to enter. However, cells with damaged membrane uptake the dye and stain positive. Cellular viability assays were conducted 35 h following the infections.

### NF-κB Transcription Induction Experiments

A549 cells were infected with AdNFkBLuc construct at an MOI of 5000 DNA particles/cell to determine the NF-κB activation status. AdNFkBLuc vector carries four tandem copies of the NF-κB binding consensus sequence fused to a TATA-like promoter from the HSVTK gene. This vector has also a Luciferase reporter gene. Luciferase assays were conducted 30 h following the infection using the Luciferase assay system with Reporter Lysis Buffer as described by the manufacturer (Promega, Inc.). Bradford assay was performed to measure the protein concentration in each sample and these values have subsequently been used to normalize Relative Light Units (RLU) against the protein concentration.

### Flow Cytometry and Western Blotting

Flow cytometry assays were conducted as described previously [28]. Monoclonal antibody to TRAIL (human) (cat. no. ALX-804-296-C100; Alexis Biochemicals) was used followed by polyclonal antibody to mouse IgG1 (R-PE) (cat. no. ALX-211-201-C050; Alexis Biochemicals) to reveal TRAIL expression on the cell surface. For Western Blotting, protein extracts were prepared 48 hours following the infection. Then, 10 μg of A549 cell line extract was loaded in each lane and IKKβKA protein expression was detected using an anti-HA peroxidase antibody (Roche Molecular Diagnostic, Indianapolis, Indiana, US, Cat. No.11667475001). GAPDH expression was detected using a GADPH antibody (BIODESIGN International, Maine, US, Cat No. H86504).

### Confirmation of apoptosis induction by Annexin V staining using flow cytometry

FITC-conjugated human Annexin V (ALX-209-250-T100) was used to quantitate the number of apoptotic cells using flow cytometry. Annexin V staining procedure was performed according to manufacturer's protocols (Alexis Biochemicals).

### Caspase Activity Assays

It is well established that carboxyfluorescein-labeled caspase inhibitors can irreversibly bind to active caspases. The caspase inhibitor substrates were designed to be not only specific for the active state of the enzyme and also it is isoform specific. CaspaTag Caspase Activity Kits were deployed to selectively monitor caspase activation following infection with gene therapy vectors. FAM-DEVD-FMK (S7301) was used to measure caspase 3 activation, and then distinguished caspase positive cells from caspase negative cells by immune fluorescence microscopy.

## Results

### Efficient adenoviral transduction of A549 lung cancer cells

**Figure 1:**
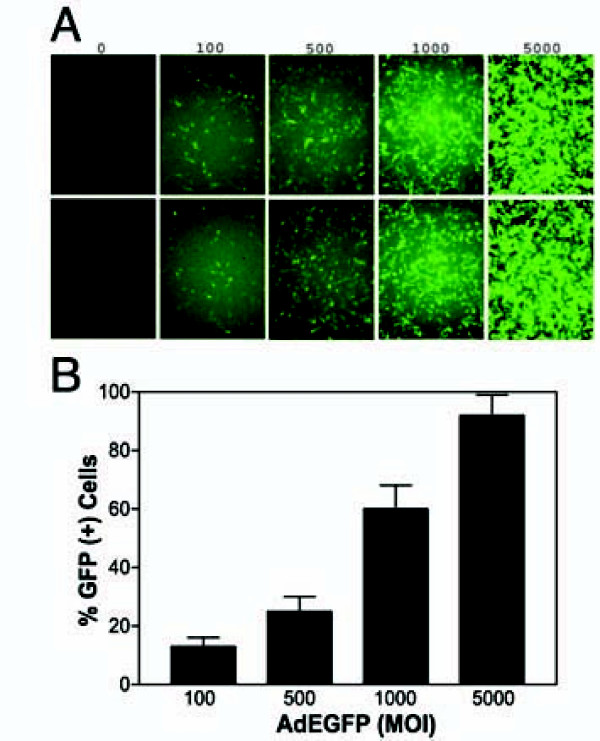
**Efficient transduction of A549 cells with recombinant adenovirus**. A549 cells were infected with increasing MOI of adenovirus encoding the EGFP reporter gene for 48 h. EGFP positive cells were detected by fluorescent microscopy (Panel A), and then analyzed by flow cytometry (Panel B). Viral doses applied as MOI values in DNA particles/cell are indicated.

The efficacy of recombinant adenoviral vector transduction of A549 lung cancer cells was revealed using an AdEGFP vector to determine the optimum dose of adenovirus needed to conduct gene delivery, which is mainly influenced by viral preparations. While fluorescent microscopy was used to follow protein expression, the transduction efficiency was quantitatively analyzed using flow cytometry 48 h following the infection. Almost 100% of the cells were efficiently transduced with AdEGFP at MOI of 5,000 DNA particles/cell 48 h after infection (Figure 1).

### A549 lung cancer cells are resistant to adenoviral delivery of hTRAIL or IKKβKA expression

**Figure 2:**
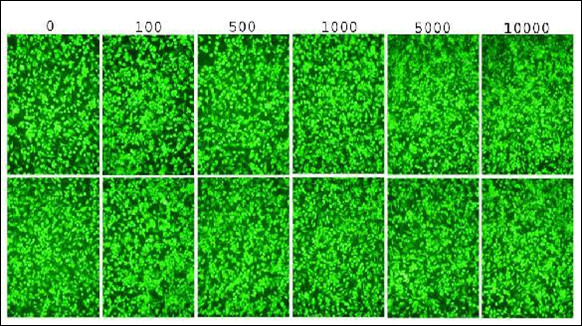
**The viability of A549 lung cancer cells is not affected by Ad5hTRAIL or AdIKKβKA infection alone**. A549 cells were infected either by Ad5hTRAIL (Upper Panels) or AdIKKβKA virus (Lower Panels) at increasing doses. Molecular Probe's Live and Death Cellular Viability and Toxicity Kit was used to detect viable cells 48 h following the infection, as described in Materials and Methods. The applied viral doses as MOI values in DNA particles/cell are indicated.

Despite the fact that TRAIL can potently induce tumor cell apoptosis, TRAIL resistance observed in some cancer cells critically challenges the use of TRAIL as a gene therapy agent. To determine the extent to which A549 lung cancer cells were susceptible to TRAIL, we infected A549 cells with an adenovirus vectors encoding hTRAIL (Ad5hTRAIL) or LacZ (AdCMVLacZ, negative control) at increasing doses and measured cellular viability following the infection. As expected, AdCMVLacZ infection alone did not reduce the number of viable cells significantly (data not shown). A549 lung cancer cells were also completely resistant to cytotoxic effects of hTRAIL, despite the high doses of Ad5hTRAIL (MOI of 10,000 DNA particles/cell) used for the infection (Figure 2, upper panels).

Increased IKK activity [29,30] and/or NF-κB activity [22] is a major regulatory obstacle against death ligand-induced cytotoxicity in various tumors. Consequently, cell survival mediated through the effect of IKK inhibition, and thereby NF-kB down-regulation, was tested after A549 infection with AdIKKβKA. As shown in Figure 2 (lower panels), no decrease in cell viability was observed even at MOI of 10,000 DNA particles/cell of AdIKKβKA vector. These results suggested that IKK inhibition alone does not affect the viability of A549 lung cancer cells.

**Figure 3:**
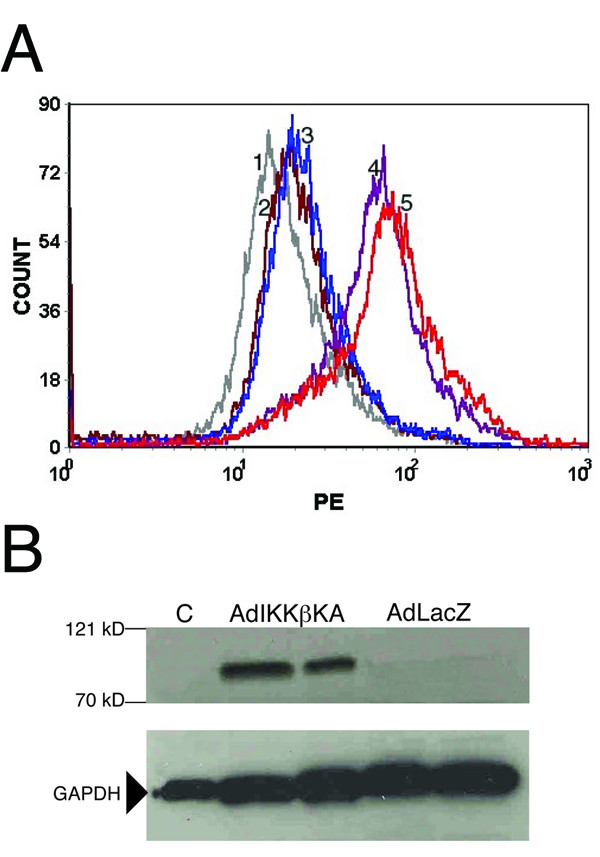
**Ad5hTRAIL and AdIKKβKA transductions of lung cancer cells**. Panel A represents a flow cytometry analysis of hTRAIL expression in A549 cell line. Conditions for infections are as follows: 1, unstained; 2, uninfected (secondary antibody alone); 3, AdLacZ; 4, Ad5hTRAIL (5,000 MOI); 5, Ad5hTRAIL (10,000 MOI). Panel B shows a Western Blotting indicating IKKβKA expression. Adenoviral constructs used in the infections are indicated above each lane (duplicate independent A549 samples are shown). Molecular standard markers (β galactosidase, 121 kD; and bovine serum albumin, 70 kD) are provided to the left of the blot.

To rule out the possibility that the lack of any cytotoxic effect was due to little/no TRAIL expression from the vector, flow cytometric analysis was performed on A549 cells infected with Ad5hTRAIL. This assay demonstrated that significant TRAIL overexpression was achieved after A549 infection with Ad5hTRAIL (Figure 3A). Similarly, immunoblot analysis was employed to demonstrate IKKβKA expression. IKKβKA expression was detectable only when cells were infected with AdIKKβKA vector but not with AdCMVLacZ (Figure 3B).

### NF-κB blocking via IKK inhibition sensitizes A549 lung cancer cells to TRAIL-induced apoptosis

**Figure 4:**
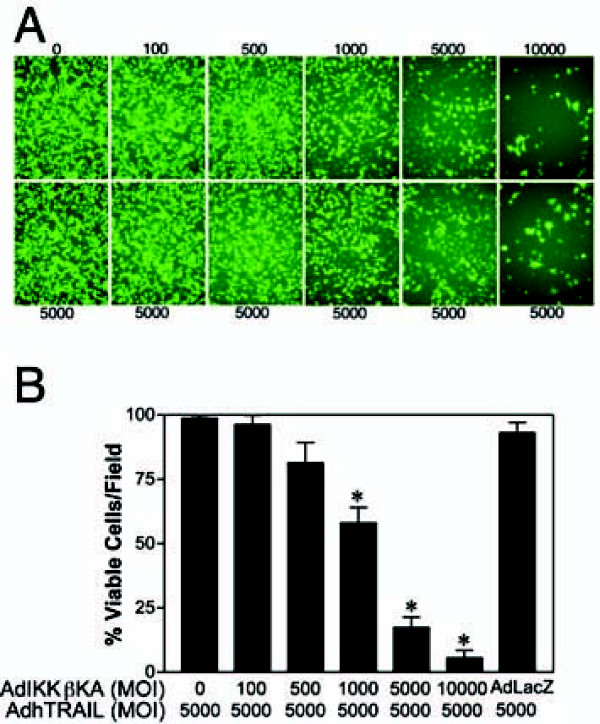
**Induction of cell death in A549 cells co-infected with AdIKKβKA and AdhTRAIL**. A549 lung cancer cells were co-infected with a constant dose of Ad5hTRAIL (5,000 MOI; as shown below each panel) and increasing doses of AdIKKβKA (as shown above each panel). Cell viability was then measured using Molecular Probe's Live and Death Cellular Viability and Toxicity Kit 48 h following infection. Panel A depicts fluorescent micrographs of such an infection. By comparison, the quantitative analysis of cell viability is provided in Panel B. Values represent the mean (± SEM) of three different experiments (n = 6). * *p *< 0.05.

Previous studies by our group have shown that A549 lung cancer cells can be sensitized to TNF by AdIKKβKA [22,24] or AdIkBαSR [26,31] expression. Since A549 cells are also resistant to TRAIL-induced apoptosis, we tested the extent to which NF-κB inhibition through IKK targetting could sensitize A549 cells to TRAIL. Thus, A549 cells were co-infected with Ad5hTRAIL (5000 particles/cell) and AdIKKβKA at increasing doses, and the percentage of viable cells was measured 48 h after infection. Over 75% cell death was observed when A549 cells were co-infected with Ad5hTRAIL and at least 5000 MOI AdIKKβKA (Figure 4). In contrast, AdCMVLacZ co-infection did not sensitize A549 cells to TRAIL. Together, these findings demonstrate that IKKβKA expression can overcome TRAIL resistance in A549 lung cells. We then tested the extent to which AdIkBαSR could substitute for the AdIKKβKA vector in sensitizing A549 cells to Ad5hTRAIL. NF-κB inhibition via AdIkBαSR infection also resulted in some degree of cell death from TRAIL, but the degree of sensitization was less than that of AdIKKβKA delivery (data not shown) suggesting AdIKKβKA inhibition of NF-κB inhibition is more efficient.

### Endogenous NF-κB activity in A549 cells is upregulated after Ad5hTRAIL infection but down regulated with IKKβKA expression

**Figure 5:**
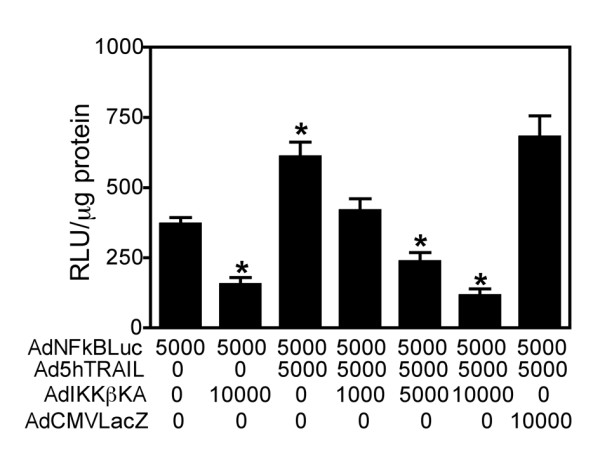
**NF-κB activity is upregulated with Ad5hTRAIL infection but down-regulated with AdIKKβKA in A549 cells**. A549 cells were simultaneously infected with AdNFkBLuc, Ad5hTRAIL, and increasing doses of AdIKKβKA construct for 30 h. As a negative control, AdCMVLacZ infection was utilized. The different constructs used in the infection and the MOI values represented in DNA particles/cell are listed on the X-axis. Luciferase activity expressed as Relative Light Units per microgram protein is depicted on the Y-axis, where the values represent the mean (± SEM) of six independent data points (n = 6). * *p *< 0.05.

Intracellular NF-κB activation is crucial for a variety of cellular functions. With respect to death ligand-induced apoptosis, high levels of NF-κB activity are associated with resistance in cancer cells [22]. In addition, signaling through DR4, DR5 [15,20], and DcR2 [8] can activate NF-κB. Thus, determining the endogenous and TRAIL-induced NF-κB activity of cancer cells before initiation of TRAIL-based therapy is important. To evaluate the extent of NF-κB activation in A549 cells, we used a recombinant adenovirus vector encoding an NF-κB driven Luciferase reporter gene (AdNFkBLuc). Luciferase expression was measured 30 h after infection. As shown in Figure 5, significant NF-κB activation was achieved after Ad5hTRAIL infection. Triple infection with AdNFkBLuc, Ad5hTRAIL and AdIKKβKA or AdCMVLacZ was then performed to check the extent to which AdIKKβKA reduced NF-κB activation. Both endogenous and TRAIL-induced NF-κB activity was drastically reduced after infection with AdIKKβKA. Conversely, AdCMVLacZ infection did not inhibit endogenous or TRAIL-induced NF-κB activity.

### Ad5hTRAIL infection with NF-κB inhibition induces apoptosis in A549 cells

**Figure 6:**
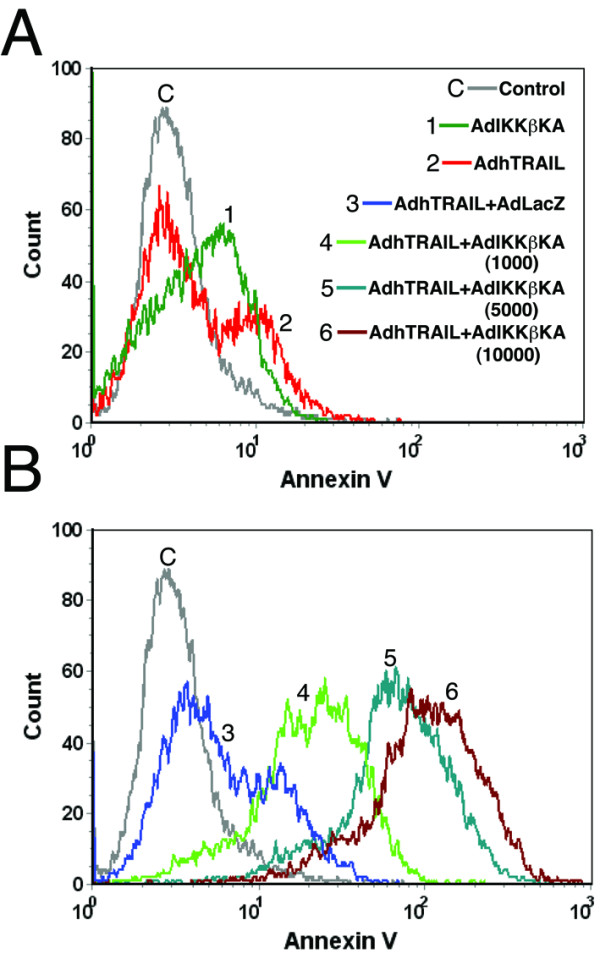
**Blocking IKK activity results in TRAIL-induced apoptosis in A549 cells**. FITC-conjugated Annexin V and Propidium Iodide (PI) staining were utilized as apoptosis indicators following infection of A549 lung cancer cells with various combinations of adenovirus constructs as stated. 10^4 ^A549 lung cancer cells were gated for each histogram. Histograms are depicted in two different panels for clarity. AdIKKβKA and Ad5hTRAIL constructs were used in 5,000 DNA particles/cell unless stated otherwise. AdCMVLacZ construct was used at an MOI of 10,000 DNA particles/cell. Uninfected but FITC-Annexin V and PI stained A549 lung cancer cells were used as controls (gray line).

**Figure 7:**
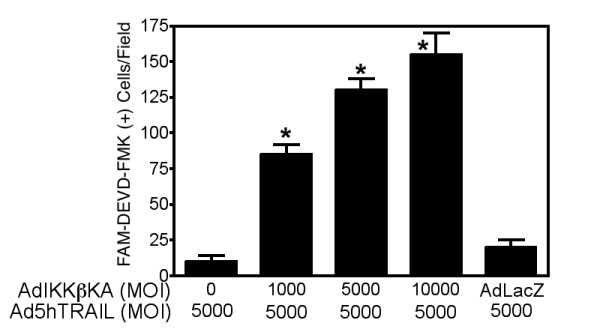
**Inhibition of IKK activity induces TRAIL-mediated Caspase 3 activation in lung cancer cells**. Caspase activity assays were performed following co-infection of A549 lung cancer cells with Ad5hTRAIL and AdIKKβKA or AdCMVLacZ vectors. MOI and types of viral vectors used in the infection are provided on the X-axis. AdCMVLacZ construct was used at an MOI of 10,000 DNA particles/cell. Caspase activity was assessed under fluorescent microscopy. Data represent the mean of (± SEM) fıve independent data points. * *p *< 0.05.

To prove that the mechanism of cell death in A549 cells following Ad5hTRAIL infection during IKK inhibition was apoptotic in nature, Annexin V staining was performed. A549 cells were infected with either Ad5hTRAIL or AdIKKβKA alone, or in combination, and apoptotic cell death was quantified 48 h following infection. There was a minimal increase in Annexin V staining on Ad5hTRAIL or AdIKKβKA infected cells (Figure 6A), but Ad5hTRAIL and AdIKKβKA coinfection resulted in a significant increase in Annexin V staining (Figure 6B). As expected, Ad5hTRAIL and AdCMVLacZ co-infection did not generate such an effect. To further demonstrate that apoptosis is the mechanism of cell death in A549 cell line, caspase activation assays were performed following coinfection of cells with Ad5hTRAIL and AdIKKβKA vectors. There was significantly increased Caspase 3 activity detected only when the A549 cells were infected with Ad5hTRAIL and AdIKKβKA (Figure 7).

## Discussion

TRAIL induces apoptosis in a wide range of malignant cells and has been heavily investigated as a potential therapeutic agent for the treatment of many tumors. These expectations were largely based on the selective apoptosis-inducing properties of TRAIL for cancer cells [32-34]. Contrary to these initial expectations, many cancer cell lines were subsequently found to be resistant to TRAIL-induced apoptosis. Consequently, a significant number of studies have been conducted to understand the molecular mechanism of TRAIL resistance in cancer cells, so this barrier could be overcome. In cancer cases where high decoy receptor expression could potentially contribute to the resistance to TRAIL, siRNA approaches have been successfully used to overcome TRAIL resistance in cancer cells, as demonstrated for breast [18], lung [28], and prostate [17] cancer cells.

Based on our previous findings and those by other groups, the NF-κB signaling pathway appeared to be one of the main molecular mechanisms responsible for the generation of TRAIL resistance in cancer cells. Overactive NF-κB activity has been implicated in many aspects of tumor formation and progression, including the inhibition of apoptosis and enhancing the expression of antiapoptotic factors [35]. NF-κB normally resides in the cytoplasm as an inactive complex with an inhibitory IκB subunit. Upon activation, IκB becomes phosphorylated by specific kinases (IκB kinase, IKK), ubiquinated, and then degraded. This inactivation of IκB enables the translocation of NF-κB into the nucleus, where it can bind to the promoter region of many genes and activate their transcription [36]. IKKβ is one of the catalytic domains of the kinase IKK and is essential for NF-κB activation. Thus, inhibition of IKKβ may be a particularly useful strategy to specifically interfere with NF-κB activity [24]. Previously, IKK targeting strategy has been successfully applied to sensitize neuroblastoma [37] and prostate cancer cells [38] to TRAIL. Although, exogenous expression of a dominant negative mutant form of IKKβ sensitized lung cancer cells to TNF by way of NF-κB inhibition, it was unknown whether this approach would similarly sensitize lung cancer cells to TRAIL. Thus, in this study we tested a complementary gene therapy modality involving IKK inhibition to overcome TRAIL resistance. In the present study, we demonstrated that inhibition of the NF-κB signaling pathway, by way of IKKβKA expression, sensitized A549 cells to TRAIL-induced apoptosis. In accordance with this, the recently identificed TRAIL receptor-binding protein, protein arginine methyltransferase 5 (PRMT5) [39], was found to potentiate TRAIL-induced NF-κB activation through IKK leading to induction of several NF-κB target genes. Interestingly, PRMT5 gene silencing sensitized various cancer cells to TRAIL. These data suggest that PRMT5 expression helped to maintain TRAIL resistance through NF-κB activation involving IKK complex in cancer cells.

## Conclusions

The IKK complex may be a good target to specifically interfere with NF-κB activation in TRAIL-resistant cancer cells, such that gene therapy strategies involving exogenous TRAIL expression with concurrent inhibition of the NF-κB pathway through IKK modulation of function may extend the therapeutic index of TRAIL for patients with lung cancer.

## Competing interests

The authors declare that they have no competing interests.

## Authors' contributions

CA and ADS designed and conducted the study. AB, LD, BK and BY helped with the assays. TSG acted as a consultant and critically reviewed the manuscript. ADS and SS supervised the study. All the authors read and approved the final manuscript.

## Pre-publication history

The pre-publication history for this paper can be accessed here:

http://www.biomedcentral.com/1471-2407/10/584/prepub
